# Analysis of the cytochrome *c-*dependent apoptosis apparatus in cells from human pancreatic carcinoma

**DOI:** 10.1038/sj.bjc.6600171

**Published:** 2002-03-18

**Authors:** M C Gerhard, R M Schmid, G Häcker

**Affiliations:** Institute for Medical Microbiology, Immunologie and Hygiene, Technische Universität München, Trogerstr. 9, D-81675 Munich, Germany; Department of Internal Medicine I, University of Ulm, Robert-Koch Str. 8, D-89081 Ulm, Germany

**Keywords:** pancreas, carcinoma, caspases, apoptosis, cytochrome *c*

## Abstract

Defects in the apoptotic system are likely to play a role in tumorigenesis. Pancreatic carcinoma cells are extremely resistant to apoptosis induction by chemotherapy suggesting that the apoptosis machinery is faulty. We investigated the integrity of the cytochrome *c*-dependent apoptotic apparatus in 10 human pancreatic carcinoma cell lines. Expression of Apaf-1, caspase-3, -6, -7, -8 and -9, Hsp-70 and XIAP was detected in all cell lines. The expression levels of Apaf-1 and caspase-8 were homogenous in all cell lines whereas differences in expression of other caspases were seen. In cytosolic fractions, all investigated caspases were processed in response to cytochrome *c* but the extent of processing varied between the cell lines. No stringent correlation between the amount of processing of caspase-9 and effector caspases was seen. Cytochrome *c*-induced effector caspase activity was quantitated by enzyme assay. Especially at low concentrations of added cytochrome *c*, this response varied greatly between the cell lines. These data demonstrate that the apoptotic system downstream of the mitochondria is qualitatively intact in pancreatic carcinoma. They further show that the response to cytochrome *c* can be quantitated in a cell-free system and that determinants other than mere expression of apoptotic molecules can regulate cytochrome *c*-induced apoptosis.

*British Journal of Cancer* (2002) **86**, 893–898. DOI: 10.1038/sj/bjc/6600171 www.bjcancer.com

© 2002 Cancer Research UK

## 

Protection against the development of cancer is probably a fundamental role of the apoptotic system (Hanahan and Weinberg, 2000). This is, on one hand, supported by the fact that experimental inhibition of apoptosis can contribute to tumorigenesis. The anti-apoptosis protein Bcl-2, for example, was able to promote the development of lymphomas in *myc*-transgenic mice (Vaux *et al*, 1988). Loss of the pro-apoptotic protein Bax has also been shown to lead to an increased appearance of tumours in a mouse tumour model (Yin *et al*, 1997). On the other hand, mutations affecting the apoptotic pathway have been found in a number of tumours. For instance, Bcl-2 is overexpressed from a chromosomal translocation in follicular lymphoma (Tsujimoto *et al*, 1985). Recent work shows that expression of caspase-8 is lost in a high number of childhood neuroblastomas (Teitz *et al*, 2000). Furthermore, the often observed loss of P53 might also contribute to a loss of sensitivity to apoptotic signals (Clarke *et al*, 1993; Lowe *et al*, 1993). Recently it was shown that cases of human melanomas have a severely diminished expression of the adapter protein Apaf-1 and are concomitantly resistant to apoptosis (Soengas *et al*, 2001). All of these points of evidence suggest that apoptosis functions as a defence mechanism against the development of cancer.

Recent research has afforded us insight into the apoptosis pathway. Central to the apoptotic signal transduction is the caspase family of proteases which are constitutively present in a cell and become activated during apoptosis. In probably all cases of apoptosis, so-called effector caspases are active. These proteases, represented predominantly by caspase-3 (probably also by caspase-6 and -7), signal apoptosis by activating catabolic enzymes and by a direct digestion of cellular proteins. The activation of effector caspases is achieved by ‘initiator caspases’ like caspase-8 and -9 (Earnshaw *et al*, 1999). Initiator caspases, in turn, are activated upon adapter-induced oligomerisation (Salvesen and Dixit, 1999). A series of reports suggest that caspase-8 and -9 control independent pathways to effector caspase-activation. While caspase-8 is activated via cell surface death-receptors and the adapter FADD/MORT1 (Fas-associated protein with death domain), caspase-9 is oligomerised and activated by the adapter Apaf-1 (apoptosis activating factor 1). Apaf-1-oligomerisation, in turn, is achieved by the binding of cytochrome *c*, and cytochrome *c-*release from mitochondria is observed in many cases of experimental apoptosis-induction.

In this study we focused on the analysis of cytochrome *c-*dependent apoptosis apparatus in cell lines derived from human pancreatic carcinomas. Pancreatic carcinoma is extremely resistant to therapeutic intervention which probably includes resistance to apoptosis induction (Warshaw and Fernandez-del Castillo, 1992). This study was undertaken with the following objectives: First, we attempted to standardise a cell-free system. Since the induction of apoptosis by exogenously added substances depends on a great number of determinants, for instance mechanisms governing uptake and cellular receptors, we used this cell-free system to analyse specifically the caspase-activating capacity of cytochrome *c* in cell extracts. Second, chemotherapeutic agents probably act at least in part to induce apoptosis via cytochrome *c-*release (Debatin, 2000). The poor response of pancreatic carcinoma to chemotherapy suggests that the cancer cells are resistant to induction of apoptosis by these drugs. Likewise, only two out of five pancreatic carcinoma cell lines underwent strong (and one intermediate) apoptosis upon viral delivery of the fragile histidine triad (FHIT) gene *in vitro* indicating a central apoptosis defect at least in some cases (Dumon *et al*, 2001). We therefore aimed at an analysis of the central cytochrome *c-*driven apoptosis pathway in cell lines derived from pancreatic carcinomas. The comparison of a number of cell lines further allowed conclusions whether expression of Apaf-1 and caspases directly correlates with the cytochrome *c-*elicited response or whether further regulatory mechanisms are in place in pancreatic carcinoma.

## MATERIALS AND METHODS

### Cell lines and culture conditions

Pancreatic carcinoma cell lines AsPC-1 (ATCC, CRL-1682), BxPC-3 (Tan *et al*, 1986), MiaPaCa-2 (ATCC, CRL-1420), Panc-1 (ATCC, CRL-1469), PaTu8988t (differentiated) and PaTu8988s (undifferentiated, metastases forming) (these two lines are from the same patient (Elsasser *et al*, 1993)) were cultured in Dulbecco's Modified Eagle Medium with 10% FCS; Capan-1 (ATCC, HTB-79), SW850 (Schmiegel *et al*, 1993) and SW-979 (Kalthoff *et al*, 1993) in Click's RPMI Medium with 10% FCS. Cells were seeded in tissue culture dishes and cultured at 37°C for 3–5 days, harvested in sub-confluent state and lysed as described below.

### Extract preparation

Adherent cells were washed in phosphate buffered saline (Biochrom), trypsinised, centrifuged and resuspended in hypotonic lysis buffer (S100 buffer: 20 mM HEPES-KOH pH 7.5, 10 mM KCl, 1.5 mM MgCl_2_, 1 mM Na-EDTA, 1 mM Na-EGTA) with a protease inhibitor cocktail (Roche) and 2 mM dithiothreitol (DTT). For extraction the suspension was incubated for 15 min on ice. Cells were then lysed by passaging them five to 20 times through a 22 gauge needle until about 80 to 100% of the cells were open as assessed by eosin exclusion. The lysate was then centrifuged at 10 000 *g* and the supernatant from this step (cytosolic fractions) was immediately frozen in aliquots at −70°C. Before use extracts were thawed on ice and total protein content was estimated by measuring absorption at 280 nm in comparison to a BSA standard.

### Caspase activation by cytochrome *c*

Caspase activation was assessed after incubation of the cytosolic extracts in S100 buffer with defined amounts of total protein with various concentrations of bovine heart cytochrome *c* (Sigma) in the presence or absence of 1 mM dATP and in presence of 1 mM DTT. Effector caspase activity was determined after 1 h of incubation at 37°C by measuring the extent of cleavage of the synthetic fluoro genic substrate acetyl-aspartate-glutamate-valin-aspartate-7-amino-4-methyl-coumarin (Ac-DEVD-AMC) (Bachem; fluorescence of free AMC: excitation 360 nm, emission 460 nm) in a Millipore Cytofluor 2350 Fluorescence Measurement System at a series of time points after addition of substrate as described (Linsinger *et al*, 1999). A linear regression was established of the linear increase of the fluorescence during the first 10–20 min after addition of Ac-DEVD-AMC. The regression coefficient was defined as effector caspase activity.

### Western blotting

Expression of apoptosis-relevant proteins and processing of pro-caspases was measured by Western blotting. Samples were subjected to SDS–PAGE, transferred to a nitrocellulose membrane and proteins were detected using specific antibodies and horseradish peroxidase conjugated secondary antibodies (antibodies were from Pharmingen (anti-caspase-3, caspase-6, caspase-7, caspase-9 and Apaf-1), Upstate Biotechnologies (anti-caspase-8), Stressgen (anti-Hsp-70 (heat shock protein 70)) and R&D Systems (XIAP (X-linked inhibitor of apoptosis protein))).

## RESULTS

### Pancreatic carcinomas vary in expression of components of the apoptotic system and in the propensity to process** caspases in response to cytochrome *c*

**Figure 1:**
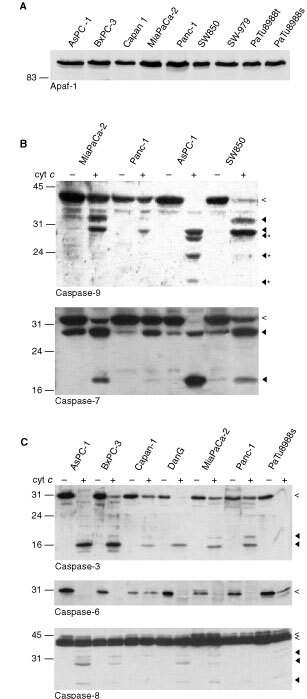
Expression of core components of apoptosis in pancreatic carcinoma cell lines. Expression of Apaf-1, of caspase-3, -6, -7, -8, -9 and cytochrome *c-*induced processing of caspase-3, -6, -7, -8 and -9 were assessed by Western blotting. Extracts (200 μg total protein) were subjected to SDS–PAGE either directly (**A**) or after 1 h of incubation at 37°C (20 mg ml^−1^ in **B** and **C**; 10 μl were loaded for Western blot analysis) in the absence or presence of cytochrome *c* (50 μg ml^−1^) as indicated. Western blot analysis was performed using antibodies specific for Apaf-1 (**A**), caspase-9 (**B**, top), -7 (**B**, bottom), -3 (**C**, top), -6 (**C**, middle) and -8 (**C**, bottom). Open arrowheads show the pro-caspase, filled arrowheads the processed forms of caspases. Cyt *c*: cytochrome *c*; *Unusual processed form of caspase-9 in AsPC-1. We were unable to detect processed caspase-6 with the antibody used since this antibody showed strong cross-reactivity with cytochrome *c* (which ran at about the same molecular size, not shown).

The central signal transduction complex of cytochrome *c-*dependent apoptosis encompasses the proteins Apaf-1, caspase-9, caspase-3 and perhaps further components such as Hsp-70 and XIAP (Beere *et al*, 2000; Saleh *et al*, 2000; Bratton *et al*, 2001). We tested expression of these components by Western blotting. Processing of caspases upon incubation with dATP/cytochrome *c* was further investigated. For most cell lines, between two and four different preparations of extract were tested. Figure 1

details examples of the experiments.

The expression of caspase-8 and Apaf-1 was homogenous across all cell lines tested (Figure 1A,C bottom and data not shown). All the caspases investigated were detectable in all cell lines although there was variation in expression level between the lines. The expression levels of caspase-3, -6, -7 and -9 were comparable in all cell lines (Figure 1B,C and data not shown). Relatively low expression of caspase-6 was observed in the cell lines Capan-1 and MiaPaCa-2 (Figure 1C and data not shown).

To assess the capacity for caspase processing, cell extracts were subjected to Western blotting upon incubation at 37°C in the presence of dATP and either without or with cytochrome *c*. Cytochrome *c* activates apoptotic signaling by binding to Apaf-1 which in turn forms (in the presence of dATP) high molecular weight complexes consisting of several molecules of Apaf-1, caspase-9 and caspase-3. In the course of this association, first caspase-9 and later caspase-3 is activated (Cain *et al*, 1999; Rodriguez and Lazebnik, 1999; Zou *et al*, 1999; Bratton *et al*, 2001). Examples of results from these experiments are given in Figure 1B,C. A summary of the semi-quantitative evaluation of the results is given in Table 1

**Table 1:**
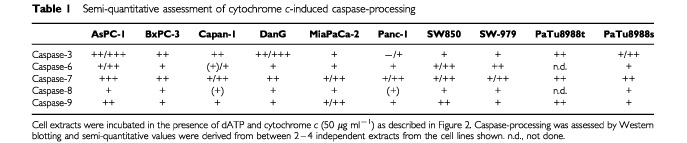
Semi-quantitative assessment of cytochrome *c*-induced caspase-processing

.

Intriguingly, despite the relatively even expression levels between the cell lines, we found pronounced differences in cytochrome *c-*induced caspase-processing. Processing of the apical caspase caspase-9 was seen in all cell lines, strongest in the lines AsPC-1 and SW850 (Figure 1B). In extracts only from AsPC-1 cells bands were detected by Western blotting which corresponded to even smaller proteins than the 35 and 37 kD processed forms described (Jiang and Wang, 2000); in the other cell lines exclusively the known processing products were seen. As far as we are aware, such smaller processing bands have not been described before; they appear to result from further proteolytic processing, possibly related to caspase-activity. It is remarkable that – although saturating concentrations of cytochrome *c* were used in these experiments (see below) – differences in the activation of caspase-9 were apparent between the cell lines. This indicates that expression of the core proteins Apaf-1 and caspase-9 is not the only determinant of this activation.

**Figure 3:**
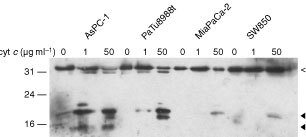
Processing of caspase-3 by two concentrations of cytochrome *c*. Extracts from the cell lines AsPC-1, PaTu8988t, MiaPaCa-2 and SW850 were incubated in the absence or the presence of either 1 or 50 μg ml^−1^ cytochrome *c* as described above. After 1 h, samples were taken and subjected to Western blotting with antibodies specific for caspase-3.

The processing of caspase-3 should in the simplest case be directly correlated with the processing of caspase-9. We did, however, not find the expected stringent correlation. While in AsPC-1-extracts the caspase-3-processing paralleled the strong caspase-9-processing, MiaPaCa-2 and SW850 showed only moderate processing of caspase-3 in the presence of stronger caspase-9-activation indicating that further regulatory factors of this step exist. In support of this view, the line DanG showed only weak processing of caspase-9 but strong processing of caspase-3 (Table 1, Figures 1B,C and 3

).

Caspase-6 and caspase-7 are ranked as effector caspases which might fulfill independent functions in cell death: they are activated in response to caspase-9-activation although it is not entirely clear whether directly or upon activation of caspase-3 (Slee *et al*, 1999; Vier *et al*, 2000). Caspase-6 was processed upon addition of cytochrome *c* although to different extents (Figure 1C). The same was the case for caspase-7 (Figure 1B).

Caspase-8 is normally activated upon death receptor signalling, following recruitment to the plasma membrane (Kischkel *et al*, 1995). It can, however, also be activated secondarily to effector caspase-activation during drug-induced apoptosis but the significance of this activation is unknown (Ferrari *et al*, 1998). In all lines a slight processing of caspase-8 upon cytochrome *c-*addition was detected. This processing correlated with the processing of caspase-3 and is likely to be the result of ‘retrograde’ activation of caspase-8 by caspase-3 (Figure 1C and see below).

### Induction of effector caspase-activity by cytochrome *c*

Effector caspases will lead to apoptosis by their proteolytic activity cleaving after the tetrapeptide DEVD. This activity is probably mainly conveyed by caspase-3, to a lesser extent by caspase-7 (Thornberry *et al*, 1997; Earnshaw *et al*, 1999). The generation of this DEVD-cleaving (effector caspase) activity was measured in extracts from the pancreatic carcinoma cell lines upon incubation with cytochrome *c*. For a quantitative assessment of the activity, DEVD-cleaving activity was calculated by linear regression analysis as the regression coefficient of free AMC fluorescence under conditions of substrate saturation. Titration of the concentration of cytochrome *c* showed that concentrations above about 25 μg ml^−1^ yielded maximal caspase-activation (‘saturating concentration’; data not shown). Caspase-activity was consecutively compared between the cell lines at saturating concentrations of cytochrome *c* (50 μg ml^−1^) and at one lower concentration (1 μg ml^−1^).

**Figure 2:**
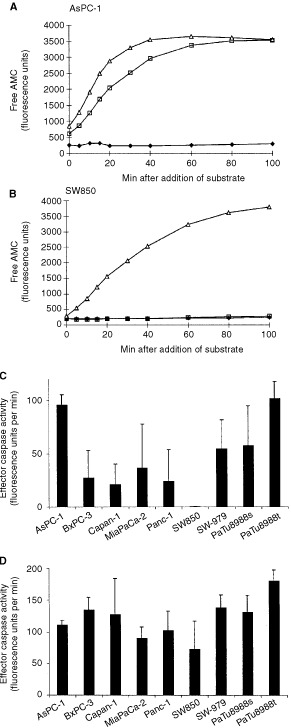
Induction of effector caspase activity by cytochrome *c*. Cell extracts (20 mg ml^−1^ total protein) were incubated with 1 mM dATP for 1 h at 37°C in the absence or presence of cytochrome *c*; then the fluorogenic substrate DEVD-AMC was added. The release of fluorescent AMC was monitored over a period of 100 min. (**A**), extract from AsPC-1, (**B**), extract from SW850. Curves show the activity generated in the absence of cytochrome *c* (filled squares) and in the presence of 1 μg ml^−1^ (open squares) or 50 μg ml^−1^ (open triangles) of cytochrome *c*. (**C**,**D**) Three independently prepared extracts from each cell line were incubated for 1 h at 37°C (20 mg ml^−1^ total protein) in the presence of either 1 μg ml^−1^ (**C**) or 50 μg ml^−1^ (**D**) cytochrome *c*. Results are presented as the mean and standard error of the mean of the regression coefficient determined as described in Materials and Methods.

At saturating concentrations of cytochrome *c* extracts of all tested pancreatic carcinomas showed increased effector caspase activity compared with the control without cytochrome *c*. The DEVD-AMC-cleaving activity depended on the amount of cytochrome *c* added and this sensitivity towards cytochrome *c* was greatly different between the cell lines. Differences in this response were seen at saturating concentrations (Figure 2D

) but were even more pronounced at low concentrations of cytochrome *c* (Figure 2C). Extracts from the cell line SW850 (Figure 2B,C) were almost completely refractory to low cytochrome *c-*concentrations while AsPC-1 (Figure 2A,C) and PaTu8988t-extracts (Figure 2C) showed already almost maximal caspase-activation. The other lines were placed between these two extremes, again with significant differences in sensitivity. A summary of the experiments with all lines is shown in Figure 2C,D.

Since this response did not correlate with expression of either Apaf-1, caspase-9 or caspase-3 (compare e.g. AsPC1, SW850 and MiaPaCa-2 as to their expression (Figure 1) and the elicited activity (Figure 2 and Table 1)), these results suggest that further determinants exist in these cells which govern caspase activation by cytochrome *c*.

### Cytochrome *c*-induced effector caspase activity correlates with the degree of caspase-3 processing

Effector caspase activation measured as DEVD-AMC cleavage was in further experiments correlated with the processing of caspase-3. For instance, in AsPC-1 extracts (which showed strong DEVD-AMC cleaving activity when incubated with cytochrome *c*), strong processing of caspase-3 was detected (Figure 3) while only poor processing of this caspase occurred in extracts from the cell lines SW850, MiaPaCa-2 and Panc-1 in good correlation with the low levels of effector caspase activity measured.

Caspase-3 activity is probably responsible for at least a majority of the observed morphological and functional alterations during apoptosis, for instance the well-characterized nuclear changes (although apoptosis proceeds normally even in the absence of a nucleus (Jacobson *et al*, 1994)). We established that staurosporine was able to induce a varying degree of nuclear degradation (‘laddering’) in the four cell lines investigated (AsPC-1, MiaPaCa-2, SW850 and BxPC-3) indicating that this part of the apoptotic machinery is also intact (data not shown).

### Expression of the potential regulators of cytochrome-*c*-dependent caspase-activation, Hsp-70 and XIAP

**Figure 4:**
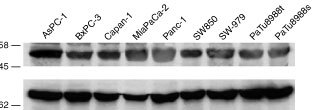
Expression of Hsp70 and XIAP in pancreatic carcinoma cell lines. Samples of extracts from the indicated cell lines (200 μg total protein/lane) were subjected to Western blotting with antibodies specific for XIAP (top) or Hsp70 (bottom).

Heat shock protein 70 (Hsp70) (Beere *et al*, 2000; Saleh *et al*, 2000) and the X-linked inhibitor of apoptosis protein (XIAP) (Deveraux *et al*, 1997) were shown to be able to inhibit cytochrome *c-*dependent caspase-activation; XIAP furthermore co-elutes with the active Apaf-1/caspase-9/caspase-3-complex (Bratton *et al*, 2001). As shown in Figure 4

, all cell lines expressed these two proteins to a similar level. The minor differences detected did not correlate with the sensitivity towards cytochrome *c*. Therefore, a regulation of cytochrome *c-*induced caspase-activation by these two factors is not evident in human pancreatic carcinoma.

## DISCUSSION

We investigated the central signal transduction of apoptosis in cell extracts from pancreatic carcinoma cell lines. The results show that all known essential components of this pathway were expressed in all cell lines. There were, however, differences between the cell lines in effector caspase-processing and cytochrome *c-*induced activity. The comparison between a number of cell lines further allows conclusions about regulatory steps in the cell death pathway.

Since the discovery that free cytosolic cytochrome *c* can activate caspases (Liu *et al*, 1996), numerous examples have been reported demonstrating that cytochrome *c* released from mitochondria can trigger caspase-activation in cytosolic extracts. To our knowledge, however, no system has been established which would allow the quantification of caspase-activation in cell extracts. Our results show that it is indeed possible to obtain reproducible results which permit the assessment of a relative sensitivity towards cytosolic cytochrome *c*.

All pancreatic tumour cell lines investigated expressed all the known core components, although not in all cases to the same levels. A number of recent studies have investigated the expression of these proteins in various types of tumour cells and reported differences, for instance loss of caspase-8-expression in a number of neuroblastomas and of Apaf-1 in melanomas (Teitz *et al*, 2000; Soengas *et al*, 2001). In other tumour types such as acute leukaemia these proteins appear to be generally expressed but at widely different levels without a clear correlation with the response to chemotherapy (Svingen *et al*, 2000). The expression of some components in the pancreatic cell lines was found to be remarkably similar, especially of caspase-8 and Apaf-1 but also, with some small variations, of caspase-9. The strongest variation was found for caspase-6, second for caspase-3.

The available data regarding expression of caspases in tissue are limited since most studies have been carried out on tumour cell lines. In the case of the pancreas, it has been suggested that expression of caspase-1 is elevated in adenocarcinoma as compared to normal pancreas tissue (Gansauge *et al*, 1998). Caspase-1 is, however, usually most likely not involved in cell death and therefore an unlikely regulator of apoptosis. Another study found a higher level of caspase-3 mRNA in pancreatic carcinoma than in normal tissue (Satoh *et al*, 2000). How this elevated gene expression could be correlated with malignancy is unclear. We compared expression levels in the pancreatic carcinoma cell lines with the expression of the well-studied T-cell line Jurkat. Expression of caspases in Jurkat cells is comparable to the level seen in normal human T-cells and was found to be in the same range as in pancreatic carcinomas (data not shown). Changes in expression of caspases are therefore unlikely to contribute to tumorigenesis of pancreatic carcinomas.

The expression of Apaf-1 and caspase-8 was surprisingly even in all tested cell lines. Little is known about the regulation of expression of these proteins; our data suggest that the regulation of expression of some caspases (especially caspase-8 and -9) is more stable than and therefore regulated differently from others such as caspase-3 and -6. The investigation of a number of cell lines enabled us to compare the events of caspase-expression, proteolytic activation and activity and assess correlations (or lack of correlation) within these entities. The simplest perception is that cytochrome *c* allows binding of dATP to Apaf-1 which in turn permits the formation of a complex containing Apaf-1, caspase-9 and caspase-3 (Cain *et al*, 1999). Formation of this complex leads to the activation of caspase-9 and caspase-3. This process can indeed be re-played using the purified components (Jiang and Wang, 2000). However, analysis of extracts from pancreatic tumour cell lines shows that this process is not as straightforward as these data suggest. We did observe a correlation between the proteolytic activation of caspase-3/caspase-7 and the detectable DEVD-cleaving activity. There was, however, no clear correlation between the measured DEVD-cleaving activity and the level of expression of either caspase-3, -7 or -9. Furthermore, the amount of processing of caspase-9 was not stringently correlated with the processing of caspase-3. Finally, the response to the addition of cytochrome *c* (as measured as effector caspase-processing and DEVD-cleaving activity) varied between the different cell lines (especially at 1 μg ml^−1^ cytochrome *c*) despite the relatively even expression of Apaf-1 and caspase-9. These results imply that regulatory steps exist other than mere expression which govern this activation. Expression of two candidate regulator proteins, Hsp70 and XIAP, did not provide an explanation: cell lines with poor response to cytochrome *c* did not express higher levels of these inhibitory proteins than good responders. It is therefore likely, that further, unknown regulatory components of the cell death pathway exist which are able to impact at this central point of apoptosis transduction.

In summary, these data attest the general integrity of the apoptosis pathway downstream of mitochondria in all 10 investigated cell lines from human pancreatic carcinoma. The relatively poor response to cytochrome *c* in some lines (such as SW850 or Panc-1) might have contributed to tumour development, taking into account that tumour biology probably depends on a subtle balance of apoptosis-inducing and -inhibiting factors *in vivo*. It is, however, unlikely from our data that defects in the apoptotic core-system are the main reason for the apoptosis-resistance of these cells. The investigated cell lines have various genetic make-ups: for instance, BxPC-3 is unusual in that it contains an intact P53 protein, and SW850 and SW-979 have a normal K-ras protein; the similar pattern of reaction in all lines indicates that the frequently observed mutations in these oncogenes do not necessarily lead to alterations in the central apoptosis pathway. Apoptosis resistance might be correlated to changes of upstream apoptosis regulators such as the Bcl-2-family of proteins. It is, however, important to note that future attempts at reversing an apoptosis resistance in pancreatic carcinomas should focus on such upstream parts of the apoptosis pathway and that therapeutical correction of the downstream parts is probably not required.
